# Perceptions and Use of Clinical Practice Guidelines in Psychosocial Oncology—A Pan-Canadian Survey of Mental Health and Social Service Professionals

**DOI:** 10.3390/curroncol33070380

**Published:** 2026-06-24

**Authors:** Catherine Bergeron, Carmen G. Loiselle, Martin Drapeau, Annett Körner

**Affiliations:** 1 Department of Educational and Counselling Psychology, McGill University, Montreal, QC H3A 1X1, Canada; martin.drapeau@mcgill.ca (M.D.); annett.korner@mcgill.ca (A.K.); 2 Ingram School of Nursing, McGill University, Montreal, QC H3A 0G4, Canada; carmen.g.loiselle@mcgill.ca; 3 Gerald Bronfman Department of Oncology, McGill University, Montreal, QC H4A 3T2, Canada; 4 Lady Davis Institute for Medical Research, Jewish General Hospital, Montreal, QC H3T 1E2, Canada; 5 Cancer Research Program, McGill University Health Centre, Montreal, QC H4A 3T2, Canada

**Keywords:** evidence-based practice, psychosocial oncology, clinical practice guidelines, implementation

## Abstract

Rising cancer incidence and survival rates have led to an unprecedented demand for psychosocial care. Yet, limited financial and practical resources present a barrier to the provision of evidence-based care. Clinical practice guidelines (CPGs) are well-positioned to enhance the quality and efficiency of psychosocial oncology care; however, little is known about their use and perceptions in the field. The present study explored the use and perceptions of CPGs among 172 Canadian psychosocial oncology clinicians via a cross-sectional, online survey. Findings revealed substantial variation in awareness, with over 20% of participants reporting no familiarity with CPGs, and low to moderate use of CPGs (*M* = 2.97, *SD* = 2.96) among users. Key barriers included a lack of formal training, limited applicability to local contexts, and systemic constraints such as high workloads. Conversely, participants highly endorsed facilitators, including accessible training programs, relevant tools/interventions, and greater institutional and community engagement. Clinician perspectives are paramount to the dissemination and implementation of psychosocial oncology CPGs. Our findings suggest that successful implementation requires broader accessibility, widespread adaptation, and greater community engagement. By addressing these systemic constraints, CPGs may be better positioned to bridge the gap between evidence and real-world service provision.

## Simple Summary

As more people develop cancer, the demand for psychosocial support has reached an unprecedented level. Clinical practice guidelines are designed to help health professionals provide high-quality, evidence-based care, yet we know very little about how they are used in the field. In this study, we surveyed 172 Canadian clinicians to understand their perspectives on using these guidelines. We found that awareness varies significantly; over 20% of clinicians were unfamiliar with existing guidelines, and those who were aware reported only limited use. Key challenges included a lack of formal training, high workloads, and difficulty applying guidelines to local settings. However, clinicians believe that better access to training and stronger institutional support would help. These insights are essential for bridging the gap between research and practice, ensuring that psychosocial oncology guidelines are successfully integrated into real-world cancer care.

## 1. Introduction

Cancer represents a major global health burden, with more than 19.3 million new diagnoses each year, accounting for approximately one-sixth of all deaths worldwide [1]. In Canada alone, an estimated 247,100 individuals are expected to receive a diagnosis, and as many as 88,100 cancer-related deaths are expected annually, leading to profound individual and systemic burdens [2]. Beyond physical symptoms and treatment-related side effects, patients frequently face significant psychological, functional, social, and practical challenges, such as emotional distress, depression, anxiety, fear of recurrence, sexual dysfunction, disturbances in social and daily functioning, and existential distress [3,4,5]. These multidimensional issues are present regardless of tumor type or stage at diagnosis, with many individuals continuing to report psychosocial difficulties well into survivorship and particularly after the end of their active treatment phase [5,6,7,8]. The timely and efficient provision of psychosocial care when needed is especially important as unaddressed distress can interfere with treatment adherence, recovery, and overall quality of life [9,10,11].

A landmark report released by the Institute of Medicine (IOM, renamed to National Academy of Medicine (NAM) in 2015) highlighted psychosocial care as an essential component of comprehensive cancer care, reflecting a growing awareness of the importance of addressing psychological symptoms to optimize patient care and outcomes [12]. Accordingly, substantial global investments in the field further reflect this growing recognition, with over $523 million USD awarded to psychosocial oncology research between 2016 and 2020 [13]. Consequently, these investments have led to a rapidly expanding evidence base, with a wide range of reliable, empirically validated psychological interventions [14,15]. Yet, this investment represents a small portion of overall funding in cancer research, suggesting that the psychosocial oncology field remains neglected despite growing evidence for its significant benefits [13]. More recently, the American Society of Clinical Oncology (ASCO) and American Association for Cancer Research (AACR) raised concerns about recent U.S. federal budget cuts to cancer research [16,17]. Moreover, patient-reported outcomes continue to show significant unmet psychosocial and communication needs [18,19,20,21], and clinicians report persistent gaps in care delivery. Routine distress screening and referrals to appropriate services, for instance, are often inconsistently implemented [22,23,24].

On a more positive note, psychosocial oncology research is being generated at an unprecedented speed. A bibliometric analysis generated between 1980 and 2021 shows a steep increase in published studies since 2010, with more than 365 relevant articles published in 2021 alone. However, this growth also represents a significant challenge for clinicians, as they would need to read one new article per day to remain fully up-to-date [25]. Coupled with increasing clinical demands due to rising cancer incidence rates and survivorship, it seems impossible for clinicians to read, evaluate, and integrate the latest research into their clinical practice. Not keeping up with relevant research to guide clinic practice may lead to variability in service delivery and patient outcomes [25,26]. A systematic review assessing barriers to the provision of evidence-based psychosocial oncology care further supports this notion; clinicians frequently reported obstacles such as workload pressures, time constraints, insufficient training opportunities, and uncertainty about the credibility or potential harms of new interventions [27]. Together, these factors highlight a critical need for a way to facilitate the translation of research evidence into practice without further burdening clinicians.

### 1.1. The Role of Clinical Practice Guidelines in Bridging the Implementation Gap

Clinical practice guidelines (CPGs) are well-placed to address this need. They are defined by the U.S. Institute of Medicine as “…*statements that include recommendations intended to optimize patient care that are informed by a systematic review of evidence and an assessment of the benefits and harms of alternative care options*” [28]. In other words, CPGs serve to systematically identify and synthesize the latest scientific findings into specific, actionable recommendations for clinical care that consider the potential harms and benefits of different treatment options. CPGs are designed to guide the decisions of healthcare professionals, reduce variability in service delivery, and improve efficiency, quality, and equity of services [29]. In medicine, failure to follow CPGs has been linked to suboptimal treatment, medication errors, and unneeded or harmful interventions [30,31,32,33]. In oncology specifically, CPG adherence has been associated with improvements in overall survival rates [34].

Within psychosocial oncology, numerous CPGs are available to address distress, anxiety, depression, survivorship care, and specific symptoms such as cancer-related fatigue and sexual dysfunction. An environmental scan identified 23 up-to-date psychosocial oncology English-language CPGs internationally [35], developed by organizations such as the Canadian Association of Psychosocial Oncology (CAPO) and the National Comprehensive Cancer Network (CCO). These CPGs frequently target frontline oncology teams, composed of physicians, nurses, and social workers, as well as primary care providers (PCPs) as end users given that they deliver the vast majority of psychosocial care as part of the baseline (i.e., “universal”) care offered to patients with a cancer diagnosis [36]. At this universal tier, care primarily consists of providing informational support and targeted psychoeducation on common symptoms, such as teaching basic sleep hygiene practices to address mild insomnia [37]. However, these tools are also designed to facilitate the treatment decisions of specialized psychosocial oncology clinicians, who provide care to patients whose distress or symptoms fail to be resolved following the provision of universal psychosocial care. The present study focuses on CPG uptake among these specialized clinicians (e.g., psychologists, psychotherapists, social workers, etc.).

### 1.2. Current Understanding of CPG Implementation

Despite findings underscoring the benefits of CPG utilization, CPGs remain underutilized in healthcare [23]. A foundational systematic review by Cabana et al. [38] identified three major categories of barriers to CPG use: 1. Knowledge-related barriers (e.g., lack of awareness or familiarity, self-efficacy), 2. Attitudinal barriers (e.g., disagreement or low perceived usefulness), and 3. External barriers (e.g., workflow, institutional constraints). Findings from subsequent systematic reviews further expanded upon these findings, and proposed five implementation determinant categories: 1. Political, social, and cultural context, 2. Institutional and organizational context, 3. Guideline characteristics, 4. Clinician-related factors, and 5. Patient-related factors. Subsequent studies in psychology noted the influence of specific variables, such as high workloads, insufficient training opportunities [39,40,41], conflicting recommendations across different CPGs [26], and the perception of CPGs as being overly rigid (e.g., like “cookbooks”), prescriptive, or designed primarily to cut costs and reduce the risk of litigation rather than maximize benefits for patients [26,39,42].

In psychosocial oncology specifically, research exploring CPG implementation is scarce. A select few studies exploring the provision of CPG-concordant care in psychosocial oncology highlighted barriers such as resistance to changing established practices [43,44], time constraints, staff turnover, high workloads [43,44,45], lack of resources [44,46], poor communication among providers [43], lack of clarity in role distribution [43], and lack of training opportunities [45]. These barriers to the provision of CPG-concordant psychosocial oncology care are critical to the broader oncology audience. Frontline oncology teams seeking to provide referrals for high-needs patients frequently face significant waitlists and resource constraints, limiting their ability to ensure the provision of appropriate psychosocial care and further highlighting the importance of maximizing the efficiency of care delivery within these specialized services [47]. Research suggests that tailored, multicomponent implementation strategies such as team-based skills acquisition or online training modules may increase CPG use [48].

### 1.3. The Canadian Context and Need for the Present Study

Despite Canada’s increased investment in psychosocial oncology, CPGs, and distress screening and assessment, critical gaps remain in our understanding of Canadian clinicians’ experiences with CPGs. One systematic review exploring the perspectives of Canadian mental health clinicians more broadly highlighted a lack of awareness, resistance to changing established practices, low self-efficacy, poor institutional support, and concerns about trustworthiness as significant barriers to CPG implementation [49]. However, there is a notable lack of psychosocial oncology-specific data on CPG uptake and implementation, especially given the wide scope of clinicians targeted by CPGs [36,50,51]. As such, the specific needs and relevance of existing psychosocial oncology CPGs within Canadian contexts and healthcare structures remain largely unexplored.

**Study Objective.** The present study aims to explore the perspectives of Canadian clinicians providing specialized psychosocial oncology care as these relate to CPGs, including their use, awareness, attitudes, perceived barriers, and motivating factors for implementation.

Research Questions.

1. What is the current awareness and use of CPGs among clinicians proving psychosocial oncology care?
2. How do clinicians view the relevance, quality, and utility of these CPGs?
3. Which factors predict the use of CPGs?
4. What CPG uptake barriers and facilitators do clinicians report?

## 2. Methodology

The present study was conducted in two distinct phases consisting of the development and piloting testing of an initial survey based on an extensive literature review and expert consultation (Phase 1). Phase 2 involved a wide-scale distribution of the survey. The MEASURE Approach to Instrument Development [52] guided the development of the survey using a cross-sectional, observational approach with data collected at a single timepoint. Further details on the development process can be found in Appendix A and the Supplemental Materials.

### 2.1. Final Pan-Canadian Survey

The survey was composed of 198 items (78 required, 115 conditional, and 5 optional). Survey items were organized across four key domains: 1. Standards of practice, 2. Awareness, use, and perceptions of currently available psychosocial oncology CPGs, 3. Barriers and facilitators of CPG use, and 4. Attitudes toward evidence-based practice. More information on the survey domains and items can be found in Appendix B.

***Demographics.*** Eleven items asked about participants’ demographic characteristics including age, sex/gender, profession, place of employment, years of experience in psychosocial oncology, and proportion of time providing direct clinical care within a given week.

***Domain 1: Standards of Practice*** is composed of 19 items assessing standards of clinical practice at participants’ place of employment. Items are scored on a 5-point Likert-type scale ranging from 1 (*rarely/never*) to 5 (*always/almost always*), with higher scores indicating greater alignment with CPG-concordant care. Participants are also given the option to provide details on the standards of practice in their workplace.

***Domain 2: Awareness, Use, and Perception of CPGs*** is composed of 120 items assessing awareness of psychosocial oncology CPGs. Participants are provided with a list of all 23 existing CPGs and instructed to specify which they are familiar with. Participants are then instructed to complete three items for each selected CPG assessing use, relevance, and perceived quality. Items are scored on a 5-point Likert-type scale ranging from 1 (*rarely/never*) to 5 (*always/almost always*). Three open-ended questions ask about the use of psychosocial oncology CPGs not listed, the use of CPGs not specific to psychosocial oncology, and gaps in current psychosocial oncology CPG availability.

***Domain 3: Barriers and Facilitators to CPG Use*** is composed of 39 items assessing barriers and facilitators to CPG use. The barriers subscale consists of 26 items assessing factors that impede CPG use across four themes: 1. Knowledge, 2. Evidence and development, 3. Scope and relevance, and 4. Feasibility. The facilitators subscale consists of 13 items assessing factors that promote CPG use. Response options are scored on a 5-point Likert-type scale ranging from 1 (*strongly disagree*) to 5 (*strongly agree*). An optional open-ended question is provided after each subscale inviting participants to elaborate on barriers and facilitators.

***Domain 4: Attitudes towards Evidence-Based Practice*** is a subset of 10 items derived from the validated Evidence-Based Practice Inventory developed by Kaper et al. [53], assessing adherence to evidence-based practice (EBP). Each item was rated on a 7-point scale anchored by contrasting statements (e.g., “*I feel that EBP is useless … useful to improve my patients’ outcomes*”), with higher scores indicating more positive attitudes towards EBP as a component of clinical decision-making.

### 2.2. Participants

#### 2.2.1. Eligibility

Licensed mental health or social service practitioners providing psychosocial care to patients diagnosed with cancer in any capacity within Canada (e.g., direct service provision, leadership in a psychosocial oncology program) were eligible to participate in the survey. To assess potential variability across different professional scopes of practice and contexts, we recruited practitioners from a range of professions, including psychologists, psychotherapists, social workers, nurses, and occupational therapists who work in various settings, such as hospitals, specialized oncology programs, community centers, and private practice clinics. Eligibility was determined through two screening questions at the start of the survey.

#### 2.2.2. Participant Recruitment

Participants were recruited using multiple strategies implemented simultaneously to maximize the national reach of psychosocial oncology clinicians. Recruitment modalities included conference-based flyer distribution, targeted outreach to hospitals and community-based programs, individual outreach to clinicians identified through professional licensing bodies and online directories, and dissemination of a recruitment flyer through the newsletters of national psychosocial oncology organizations. While the availability of searchable registries varied significantly by province and professional density differed across jurisdictions, eligible clinicians from across Canada were invited to participate. A detailed description of recruitment procedures and strategies is provided in Appendix C and the Supplemental Materials.

### 2.3. Procedures

The study obtained ethical approval from the McGill University Research Ethics Board II (REB # 25-01-034). The survey was conducted remotely via Qualtrics XM software (https://www.qualtrics.com, accessed 1 January 2025) to facilitate recruitment of a representative Pan-Canadian sample. Potential participants received three e-mail messages over a four-week period containing an invitation to complete the survey through an anonymous link. Participants who completed the survey and provided their email address were sent a $20 Amazon gift card.

### 2.4. Data Analysis Plan

#### 2.4.1. Data Preparation

To minimize response bias, survey respondents who provided complete demographic data and completed at least one survey domain were retained in the analyses [54,55]. Survey responses that failed to meet these criteria were handled using pairwise deletion. As participants only rated the use, relevance, and quality for CPGs they were familiar with, participant-level means and standard deviations were calculated and used in the analysis. For 5-point Likert-type responses, options of “*Strongly Agree*” and “*Agree*” were grouped as indicators of agreement.

#### 2.4.2. Quantitative Analysis

As many variables were ordinal and several participant subgroups contained small or uneven sample sizes, non-parametric tests were used. For group comparisons, Kruskal–Wallis tests were run, with follow-up Mann–Whitney U tests. However, a Kruskal–Wallis test could not be run for comparisons across work settings as participants could select multiple applicable settings. Separate Mann–Whitney U tests were conducted for each work setting. For education level, we used three final categories (Bachelor’s/professional, Master’s, Doctoral). To control for Type I error due to multiple comparisons, Bonferroni adjustments were applied. Due to the binary coding of work settings, Bonferroni corrections for six tests yielded an adjusted significance threshold of *p* < 0.008. For education level, a Bonferroni-adjusted threshold of *p* < 0.017 was applied for three comparisons. Data analyses were conducted using IBM SPSS v. 23.

#### 2.4.3. Qualitative Analysis

Responses to open-ended questions were analyzed to complement quantitative results. Qualitative data were reviewed separately using Microsoft Excel software v. 2605 and examined using an inductive, thematic approach [56], identifying recurrent themes and illustrative insights.

## 3. Results

### 3.1. Data Cleaning and Processing

**Figure 1:**
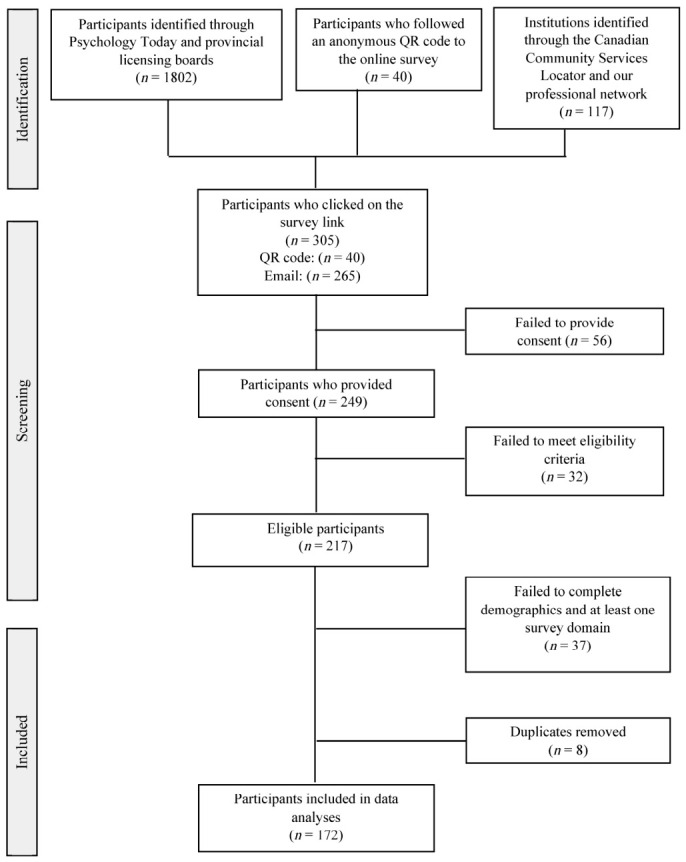
PRISMA Flow Chart for Participant Recruitment.

A total of 249 participants provided informed consent; of these, 69 were excluded as they either failed to meet eligibility criteria (*n* = 32) or did not report demographic information as well as responses to at least one survey domain (*n* = 37). Twelve duplicate IP addresses were identified. However, response patterns and timestamps suggested that 4 of these represented different participants and, thus, only eight were removed. The final sample consisted of 172 participants, of which 28 provided only partial data. The PRISMA flow chart for participant recruitment is depicted in Figure 1.

### 3.2. Descriptive Statistics

The mean age of participants was 44.85 years (*SD* = 12.44) and the sample was predominately female (88.95%). Participants were licensed across 10 Canadian provinces, with the largest percentages from Ontario (52.33%), Nova Scotia (12.21) and Quebec (11.05%). Almost all (96.58%) had a university-level or equivalent degree. The scope of professional background varied with 52 social workers (30.23%), 40 psychotherapists (23.26%), 32 psychologists (18.60%), 28 nurses (16.28%), 3 physicians (1.74%), and 2 psychiatrists (1.16%). For the purposes of data analysis, the physicians and psychiatrists were merged into the category ‘*Medical doctor*’.

Participants reported working in a variety of settings, including private practice clinics (58.14%), hospitals (31.98%), specialized cancer centers (12.21%), universities (9.88%), and community centers (7.56%). Participants tended to have significant experience working in psychosocial oncology, with 65.15% reporting >5 years. Participants reported a median range of 11 to 20 h per week dedicated to direct clinical care. See Table 1 for complete demographic information.

### 3.3. Are Current Standards of Practice Aligned with CPG Recommendations?

#### 3.3.1. Overall Standards of Practice

Across the sample (*N* = 172), participants reported that their practice follows CPG recommendations more than half of the time (*M* = 3.36, *SD* = 0.67), including screening and assessment (*M* = 3.64, *SD* = 0.92), distress monitoring and follow-up (*M* = 3.70, *SD* = 0.88), interventions (*M* = 3.22, *SD* = 0.82), and communication and care coordination procedures (*M* = 3.07, *SD* = 1.13). Item-level analyses revealed that distress screening emerged as the most frequently endorsed within the screening and assessment domain (*M* = 4.24, *SD* = 1.21). Among interventions, participants endorsed the “*Other types of empirically supported interventions*” most strongly (*M* = 3.81, *SD* = 1.20), followed by mindfulness-based stress reduction (*M* = 3.49, *SD* = 1.32), interpersonal therapy (*M* = 3.33, *SD* = 1.49), cognitive-behavioral therapy (CBT; *M* = 3.22, *SD* = 1.44), and behavioral activation (*M* = 3.11, *SD* = 1.40). Open-ended responses further highlighted routine distress screening as a standard practice within their workplace (*n* = 12, 27.28%). However, several participants reported that distress screening was not a standard procedure in their workplace due to lack of clinical staff or distress already being identified as part of the referral.

#### 3.3.2. Standards of Practice Across Different Work Settings

To compare standard practices across work settings, a Mann–Whitney U test was conducted to examine the differences in overall standards and across four practice domains between participants working in six types of work settings. Findings revealed no significant differences in overall standards of practice across the six types of work settings. However, statistically significant differences emerged for clinicians working in hospitals versus private practice clinics. Specifically, hospital-based clinicians (*n* = 55) reported significantly lower standards of practice in interventions (*M* = 2.90, *SD* = 0.86) compared to clinicians working in any other setting (*M* = 3.38, *SD* = 0.75), *U* = 2211.50, *Z* = −3.31, *p* = 0.001. However, hospital-based clinicians reported greater communication and care coordination standards (*M* = 3.51, *SD* = 0.96) compared to practitioners in other settings (*M* = 2.87, *SD* = 1.13), *U* = 2187.50, *Z* = −3.39, *p* = 0.001. Clinicians working in private practice clinics (*n* = 100) reported significantly higher standards of practice in interventions (*M* = 3.39, *SD* = 0.73) compared to clinicians working in other settings (*M* = 2.99, *SD* = 0.88), *U* = 2586.50, *Z* = −3.15, *p* = 0.002. However, clinicians in private practice reported lower communication and care coordination standards (*M* = 2.77, *SD* = 1.14) compared to clinicians in other settings (*M* = 3.50, *SD* = 0.97), *U* = 2267.00, *Z* = −4.15, *p* < 0.001. No statistically significant differences emerged for clinicians working in university or academic settings, specialized cancer centers, palliative care centers, and community centers.

#### 3.3.3. Standards of Practice by Level of Education

Kruskal–Wallis tests revealed no statistically significant differences among clinicians with different levels of education across overall standards of practice, χ2(1, 172) = 0.45, *p* = 0.798. A significant difference emerged for intervention standards (χ2(1, 172) = 8.57, *p* = 0.014), though no difference was found for screening (χ2(1, 172) = 0.33, *p* = 0.846), follow-up and monitoring (χ2(1, 172) = 1.10, *p* = 0.578), or communication and care coordination practices (χ2(1, 172) = 5.25, *p* = 0.073). Follow-up Mann–Whitney U tests revealed statistically significant differences in intervention standards among clinicians with different levels of education, with clinicians with an undergraduate or professional degree (*n* = 39) reporting significantly lower intervention standards (*M* = 2.89, *SD* = 0.90) compared to clinicians with an MA (*n* = 95; *M* = 3.32, *SD* = 0.79; *U* = 1288.00, *Z* = −2.77, *p* = 0.006) or PhD degree (*n* = 38; *M* = 3.32, *SD* = 0.72; *U* = 506.00, *Z* = −2.40, *p* = 0.016) after Bonferroni corrections for multiple comparisons. No significant differences between clinicians with MA- and PhD-level education on intervention standards were observed (*U* = 1798.00, *Z* = −0.04, *p* = 0.972).

### 3.4. Familiarity with, Use, and Perceptions of Existing PSO Guidelines

#### 3.4.1. Overall Familiarity, Use, and Perception of CPGs

Participants reported familiarity with an average of 4.21 currently existing PSO guidelines (*SD* = 4.42, range = 0–23). The majority of participants reported awareness of relatively few CPGs, with one fifth of participants reporting not being aware of any PSO guidelines and about 10% reporting familiarity with ≥10 guidelines. Among the CPGs respondents were familiar with, mean use was 2.97 (*SD* = 2.96) indicating moderate use, mean perceived relevance was 3.25 (*SD* = 3.07) reflecting substantial relevance, and perceived quality was rated as strong (*M* = 3.66; *SD* = 3.27). These participant-level averages were used for subsequent analyses comparing groups by work setting and education level. Open-ended responses highlighted positive aspects of CPGs, such as being useful in guiding their practice (*n* = 8, 42.11%), treating uncommon/new cases (*n* = 1, 5.26%), and standardizing care procedures (*n* = 1, 5.26%). However, many participants also reported limitations of the CPGs, such as being irrelevant to their practice due to the CPG’s focus or the scope of their workplace (*n* = 7, 36.84%), difficult to implement (*n* = 2, 10.53%), or unhelpful for complex cases (*n* = 1, 5.26%). Additionally, participants highlighted the need for CPGs targeting trauma/PTSD (*n* = 8, 19.05%), supportive care for family members and caregivers (*n* = 8, 19.05%), and CPG that are applicable across diverse patient cultural identities and backgrounds (*n* = 3, 7.14%), as well as the need for CPGs to consistently consider guideline implementation (*n* = 3, 7.14%).

#### 3.4.2. CPG-Level Findings

**Figure 2:**
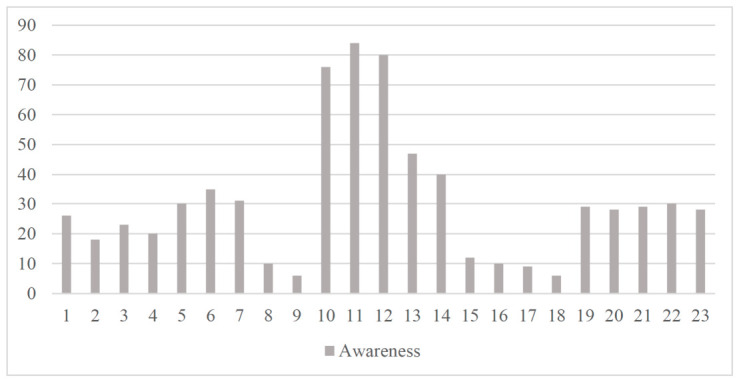
Awareness of 23 Existing CPGs Among PSO Clinicians (*N* = 172).

Clinician awareness of CPGs was generally low, with almost half of respondents reporting being unfamiliar with any of the included CPGs. Canadian CPGs had the highest levels of familiarity, with the two most frequently endorsed CPGs being ‘*Screening, Assessment and Management of Psychosocial Distress, Depression and Anxiety in Adults with Cancer*’ (*n* = 84, 48.84%) and ‘*The Management of Depression in Patients with Cancer*’ (*n* = 80, 46.51%). CPGs developed by the *National Comprehensive Cancer Network (NCCN)* and the *European Society for Medical Oncology (ESMO)* received the highest ratings for both relevance and quality. See Figure 2 for a visual representation of CPG awareness and Table 2 for a summary of use, relevance, and perceived quality ratings for each CPG.

#### 3.4.3. Perceptions and Use of CPGs Across Different Work Settings and Level of Education

No statistically significant differences in the familiarity with, use and perceived relevance and quality of CPGs were observed among clinicians in different work settings or among clinicians with different levels of education.

### 3.5. What Are the Barriers to CPG Use Among Clinicians?

#### 3.5.1. Overall Barriers to CPG Implementation

Participants endorsed a wide range of barriers limiting their use of CPGs within their clinical practice (see Table 3). Barriers related to the scope and purpose of CPGs received the highest ratings overall (*M* = 3.16, *SD* = 0.66), followed by barriers related to knowledge (*M* = 2.97, *SD* = 0.73), feasibility (*M* = 2.89, *SD* = 0.65), and development and evidence (*M* = 2.77, *SD* = 0.62). However, item-level analyses revealed substantial variations within each of these domains.

Within the ***knowledge domain***, the most highly rated items identified barriers related to difficulty knowing which CPGs are available (*M* = 3.38, *SD* = 1.06), there being too many CPGs to choose from (*M* = 3.30, *SD* = 0.94), and a lack of training on how to use and interpret CPGs (*M* = 3.26, *SD* = 1.23). Within the ***evidence and development domain***, many of the items were infrequently endorsed by participants. The most frequently endorsed barrier revealed a lack of knowledge about the quality of available CPGs (*M* = 3.22, *SD* = 1.13). Items in the ***scope and relevance domain*** were more frequently endorsed by participants, with the most frequently endorsed barriers pertaining to CPG failure to account for the local healthcare system/context (*M* = 3.49, *SD* = 0.91), neglecting to address commonly reported symptoms/concerns (*M* = 3.36, *SD* = 0.86) and not accounting for patient characteristics and preferences (*M* = 3.34, *SD* = 0.87). Finally, within the ***feasibility domain***, participants most strongly endorsed a lack of institutional support (*M* = 3.57, *SD* = 1.00) and lack of time as barriers to using CPGs (*M* = 3.09, *SD* = 1.13).

Open-ended responses highlighted several recurring themes in barriers to CPG use (*n* = 59 responses). The most frequently described barriers related to resources required for CPG uptake and implementation (*n* = 29, 49.15%) were insufficient funding, lack of staff, current workloads, and limited time to consult and implement CPG recommendations. As one participant stated: “*I am limited in my program to 4 sessions with a patient. There need to be improved guidelines to return funding to clinical support that focuses on psychological support. In the past, up to 10 sessions of support were offered… People and their situations are becoming more complex, and my work is moving to crisis management.*”. Participants also reported institutional and systemic barriers (*n* = 10, 16.95%), such as a lack of prioritization of psychosocial care, limited integration of CPGs into organizational processes, and a lack of support from management. Barriers related to knowledge of CPGs were also common (*n* = 14, 23.73%), with participants reporting limited exposure to CPGs within their training and a lack of awareness of available CPGs. Concerns about CPG characteristics were also reported (*n* = 14, 23.73%), such as perceptions that CPGs are overly generic, insufficiently tailored to patients’ needs, or limited in their scope. Finally, participants identified accessibility barriers (*n* = 8, 13.56%), such as difficulty locating CPGs, navigating websites, or being overwhelmed by the number of available CPGs.

#### 3.5.2. Barriers Across Different Work Settings

No significant differences were found in mean barrier scores among clinicians working in different settings. However, statistically significant differences emerged for barriers across the four domains. Clinicians in private practice endorsed significantly more barriers related to evidence and development (*M* = 2.89, *SD* = 0.58) compared to those working in other settings (*M* = 2.62, *SD* = 0.65), *U* = 2014.50, *Z* = −2.72, *p* = 0.007. Clinicians in private practice also endorsed significantly more barriers related to CPG scope and relevance (*M* = 3.29, *SD* = 0.56) compared to those working in other settings (*M* = 2.98, *SD* = 0.75), *U* = 2007.00, *Z* = −2.76, *p* = 0.006. No significant differences emerged within the knowledge and feasibility domains.

#### 3.5.3. Barriers Across Different Levels of Education

While no statistically significant differences were found for clinicians with different levels of education in mean barrier scores, χ2(1, 149) = 2.35, *p* = 0.309, a significant difference emerged for knowledge barriers (χ2(1, 149) = 10.21, *p* = 0.006), but not for the other 3 subdomains. Follow-up tests revealed that clinicians with a PhD-level degree reported significantly fewer knowledge barriers (*M* = 2.61, *SD* = 0.74) compared to clinicians with an undergraduate or professional degree (*M* = 3.06, *SD* = 0.73; *U* = 386.00, *Z* = −2.52, *p* = 0.012) or with an MA-level degree (*M* = 3.09, *SD* = 0.68; *U* = 865.00, *Z* = −3.08, *p* = 0.002). No significant difference in the endorsement of knowledge barriers between clinicians with an MA-level degree and those with an undergraduate or professional degree was observed, *U* = 1374.50, *Z* = −0.16, *p* = 0.876.

#### 3.5.4. Overall Facilitators to CPG Use

The mean facilitator score across the sample was 3.94 (possible range 1–5), with mean ratings across the items ranging from 3.63 to 4.11, indicating that average ratings for every item in the scale reflected overall agreement. Means and standard deviations for the reported facilitators are presented in Table 4. The following facilitators received the highest mean endorsement scores across the sample: easily accessible trainings on how to use CPGs (*M* = 4.07, *SD* = 0.92), training on using relevant tools and interventions (*M* = 4.11, *SD* = 0.86), institutional support for training opportunities (*M* = 4.11, *SD* = 1.02), easy access to recommended tools and resources (*M* = 4.07, *SD* = 0.81), and adaptations for the local healthcare system and/or context (*M* = 4.07, *SD* = 0.84). The distribution of response options signals widespread agreement with the need for these motivators to help facilitate CPG implementation across the sample of clinicians.

Open-ended responses (*n* = 35) highlighted participants’ perspectives on what would encourage CPG uptake. The most frequent answer was thorough dissemination efforts (*n* = 18, 51.43%), such as more accessible training (e.g., workshops, webinars) on CPGs and recommended interventions, access to formal continuing education programs, and the creation of a centralized repository of CPGs. Participants also emphasized the importance of actionable tools (*n* = 6, 17.14%), such as flowcharts, quick-glance summaries, decision-making aids, and standardized assessment questions. Another theme pertaining to CPG development emerged (*n* = 8, 22.86%), with participants highlighting the need for a greater emphasis on patient needs and preferences, a greater focus on cultural dimensions, consideration of approaches beyond manualized treatments, and creating adaptations for primary care providers. Participants further highlighted the need for institutional support (*n* = 8, 22.86%), such as increased funding, reduced workloads to allow time for clinicians to read and implement CPGs, and active involvement from management in dissemination and training efforts. Also, clinicians described the value of community collaboration (*n* = 9, 25.71%), noting that coordinated shared care pathways, improved communication during referral and discharge processes, and opportunities to connect with peers and colleagues help facilitate CPG implementation. Several described the importance of engagement and collaboration within the psychosocial oncology community in providing discussions about CPG recommendations, learning from each other’s experiences, and fostering shared understanding on how to apply CPGs in practice.

#### 3.5.5. Facilitators Across Different Work Settings

Mann–Whitney U tests revealed no significant difference in the overall endorsement of facilitators among clinicians in different work settings after Bonferroni corrections. Comparisons across different work settings were conducted for each individual item. Only one statistically significant difference between the facilitators emerged: clinicians in hospital settings endorsed “*openness of your institution to making changes in procedures and care standards*” more highly (Item 7: *M* = 4.02, *SD* = 1.12) compared to clinicians working in other settings (*M* = 3.44, *SD* = 1.11), *U* = 1651.50, *Z* = −3.19, *p* = 0.001.

#### 3.5.6. Facilitators Across Different Levels of Education

Kruskal–Wallis tests revealed no statistically significant differences among clinicians with different levels of education across facilitators overall, χ2(1, 148) = 1.66, *p* = 0.436, nor across each of the 12 individual facilitators.

### 3.6. Predictors of the Use of Psychosocial Oncology CPGs?

A multiple linear regression was conducted to examine whether attitudes towards EBP, perceived barriers, perceived relevance and quality of CPGs, current practice standards, years providing psychosocial oncology services, and weekly clinical hours predicted use of CPGs. The overall model was statistically significant, *F*(10, 101) = 162.65, *p* < 0.001, and accounted for a significant portion of the variance in CPG use scores, adjusted R^2^ = 0.94. Perceived relevance of CPGs emerged as the strongest predictor of CPG use, *β* = 0.83, *t*(101) = 8.67, *p* < 0.001. Overall standards of practice (*β* = 0.06, *t*(101) = 2.11, *p* = 0.037) and mean perceived barriers (*β* = 0.06, *t*(101) = 1.99, *p* = 0.049) also emerged as significant predictors of CPG use scores. In contrast, attitudes towards EBP (*β* = 0.16, *t*(101) = 0.57, *p* = 0.571), perceived quality of CPGs (*β* = 0.15, *t*(101) = 1.50, *p* = 0.138), years of experience in psychosocial oncology (*β* = 0.03, *t*(101) = 1.32, *p* = 0.191), and weekly hours of direct clinical care (*β* = −0.02, *t*(101) = −0.80, *p* = 0.427) were not significant predictors of CPG use after controlling for other variables.

## 4. Discussion

This study examined the perceptions and implementation of psychosocial oncology-specific CPGs among Canadian mental health and social service clinicians. Findings provided critical context about how these tools are currently used and have important implications for how guideline developers and stakeholders can better address the needs of clinicians. Furthermore, these findings have important implications for the global psychosocial oncology community as they offer insight into how these uptake barriers align with broader trends in implementation science beyond the Canadian healthcare context.

### 4.1. Current Use of Psychosocial Oncology CPGs

Awareness of existing CPGs varied substantially. Most clinicians were familiar with only a small subset of CPGs while over 20% were unaware of the existence of these CPGs. Of note, CPGs developed by Canadian organizations (i.e., Canadian Association of Psychosocial Oncology and Cancer Care Ontario) were the most widely recognized, lending support to the notion that adaptation to the local context may be a key determinant of CPG uptake [40,80]. While clinicians reported moderate use of CPGs, their use of these tools was largely self-driven, with participants describing selective integration of elements they perceived to be important or relevant based on their clinical expertise and organizational constraints. In fact, clinicians working in different settings may value different components of guidelines due to the differences in their professional roles and institutional capacities. Hospital-based settings are often characterized by high patient volumes, significant administrative and clinical workloads, and rigid organizational constraints, forcing clinicians to offer brief, structured interventions rather than intensive, individualized interventions; however, as they operate within multidisciplinary teams, they are exceptionally well-positioned to provide care coordination and referral services. Conversely, private practice settings often offer clinical autonomy and flexibility for highly personalized, long-term interventions, but may be professionally isolating and limit clinicians’ ability to offer multidisciplinary, coordinated treatment plans.

### 4.2. Barriers to the Implementation of Psychosocial Oncology CPGs

Three main themes emerged: 1. Knowledge and education, 2. Attitudes and perception of CPGs, and 3. Organizational constraints. These themes illustrate that fostering CPG implementation is not only a matter of individual effort on the part of clinicians but also requires wide-spread commitment from community leaders, policy makers, and healthcare institutions.

#### 4.2.1. Knowledge and Education About CPGs

Psychosocial oncology CPGs appear to be largely neglected within graduate and professional training and onboarding programs, which helps contextualize the variability in awareness. This reflects trends in healthcare more broadly, with little to no CPG education and dissemination through formal processes and clinicians highlighting the greater role of informal, peer-based efforts in promoting CPG awareness [38,39,81,82,83]. When viewed through international implementation frameworks, such as Rogers’ Diffusion of Innovations theory [84], this reliance on informal communication to diffuse awareness and knowledge of CPGs represents a well-established phenomenon rather than a challenge unique to the Canadian healthcare system. Crucially, this neglect of formal CPG training may significantly hinder the knowledge and persuasion stages of adopting an innovation (such as CPGs) and, thus, significantly hinders clinicians’ opportunities to learn about and, consequently, use these tools [38,39,81,82].

#### 4.2.2. Attitudes and Perception of CPGs

Clinician concerns about CPGs focus primarily on their relevance and clinical applicability rather than reflecting global skepticism about their utility or trustworthiness. Specifically, concerns were raised about the gaps in the symptoms targeted and the rather simplistic, rigid nature of the recommendations, which fails to address the complexity and nuance of day-to-day clinical practice. Perceived relevance and quality were key determinants in clinicians’ use of CPGs, highlighting the need for not only CPGs that are directly relevant to clinical needs but also the importance of transparent and comprehensive reporting of the development process. Interestingly, perceptions of CPG quality only partially aligned with established AGREE-II quality appraisal findings (see Chapter 3; Bergeron et al., to be submitted). However, this may reflect a lack of common understanding about how to define CPG quality, as current CPG quality assessment methods typically do not evaluate the clinical applicability and relevance of recommendations [85], while these aspects seem to be important determinants of guideline quality for clinicians. These concerns touch on a globally relevant issue pertaining to the trade-off between providing standardized, empirically supported recommendations and accounting for the complex realities of real-world clinical care.

#### 4.2.3. Organizational Constraints

Institutional procedures and a rapidly evolving healthcare ecosystem present significant challenges for clinicians as they are forced to balance their clinical responsibilities while learning and managing new technology and software, ensuring compliance with changing safety and quality regulations, engaging in administrative and supervision duties, and meeting high service demands, limitations that were frequently endorsed by participants in our sample. Participants further highlighted the impacts of limited program funding as a hinderance to clinicians’ ability to engage in continuing education and training about CPGs and to dedicate time to developing formal, routine CPG implementation procedures. Consequently, any proposed training initiatives aimed at addressing these gaps should be integrated directly into existing onboarding or training structures, with protected time provided to ensure clinicians can engage in these efforts without introducing an additional burden that further increases their workloads. Overall, these findings reflect the challenges of many healthcare systems across the world facing staff shortages, an aging workforce, and large-scale funding cuts [86,87]. Despite continually rising demands for services, psychosocial oncology remains underfunded compared to other domains of cancer care [13], further burdening existing psychosocial oncology teams and clinicians. This may have serious repercussions for distressed patients who are forced to wait for limited services and contribute to existing healthcare inequities [88].

### 4.3. Future Directions and Practice Implications

The need for formal, accessible training was consistently highlighted by clinicians as an opportunity to promote awareness and uptake of psychosocial oncology CPGs. This is consistent with findings that multi-component trainings were more effective at promoting CPG uptake in oncology settings compared to passive dissemination strategies [48], such as through the use of interactive workshops, targeted online seminars, and specialized multidisciplinary peer consultation and case discussion sessions. As such, programs embedded in professional training or onboarding offered in flexible formats present an effective solution that takes into consideration time and resource constraints. Ultimately, the systemic failure to implement and utilize these guidelines must be recognized as a form of “missed care” within oncology practice, with profound implications for patients due to high levels of distress, anxiety, and depression often remaining undetected and, thus, untreated. Beyond the consequences for patients’ quality of life, sub-optimal CPG utilization may have important financial and resource implications for the healthcare system by increasing the need for emergency and crisis services and hospitalizations [89,90]. Another key priority is accessibility. The lack of a “one-stop-shop” since the closure of the National Guidelines Clearinghouse (NGC) diminishes clinicians’ ability to easily identify and access these crucial tools. A centralized repertoire of existing CPGs would be a valuable tool not only for clinicians working in psychosocial oncology but more broadly. For example, stakeholders or institutional leaders may benefit from developing an online, open-access digital repository to host up-to-date CPGs. To ensure that this repository functions as an active clinical tool rather than a passive library, it could be directly integrated into existing workflows by embedding links to relevant CPGs within intake or other types of forms and/or screening tools. Further, the need for explicit aids and tools in applying the recommendations was highlighted within both the facilitators and barriers identified by participants. This consistent emphasis across our findings underscores the need for future guideline development to address the clinical applicability and relevance of CPGs through the greater inclusion of implementation tools (such as flowcharts, quick-glance summaries, decision-making aids, and standardized tools), flexible or tiered recommendations, nuance about diversity and cultural dimensions, and consideration of alternative, non-manualized interventions. Finally, clinicians emphasized the important role that peers and community leaders play in spreading awareness and encouraging the use of CPGs. Fostering greater community-based engagement and support within psychosocial oncology through training, supervision, and networking events and spaces creates more opportunities for clinicians to exchange with peers, learn from community leaders, conduct informal consultations, and reduce professional isolation, all of which have the potential to positively impact the ability of practitioners to engage with and implement these tools and deliver high-quality care.

### 4.4. Limitations

There were several limitations to the present study. First, survey response rates were relatively low (11%) for a survey-based study among healthcare practitioners [91,92], despite the use of incentives, suggesting that the sample may not be fully representations of the Canadian psychosocial oncology clinical population. This low response rate may stem from a lack of time, interest, or survey fatigue [93], which may be highly relevant to our population of clinicians facing high workloads and competing professional demands. Thus, the clinicians that chose to participate may be those with greater interest or engagement in guidelines, introducing a potential source of nonresponse bias. Consequently, our findings may overestimate the levels of CPG awareness and utilization among Canadian clinicians. However, the finding that numerous clinicians in our sample reported limited awareness or use of CPGs suggests that a range of perspectives was captured rather than solely representing the views of clinicians highly engaged with CPGs. Additionally, it is worth noting that defining this population is inherently challenging. Psychosocial oncology is a relatively specialized area of practice routinely practiced by only a fraction of clinicians. For example, private practice clinicians may indicate openness to treating patients with cancer but may do so only infrequently in practice and, therefore, be less inclined to participate. As such, our final sample of 172 clinicians may represent a meaningful proportion of clinicians actively engaged in this work. The final sample size remains robust according to the COSMIN Risk of Bias Checklist [94] despite this low response rate. Next, a notable shortcoming of this study is the lack of representation for frontline oncology teams and PCPs. Oncology teams are responsible for delivering universal, baseline psychosocial care, screening high-needs patients, and providing care referrals, and PCPs play a critical role in follow-up and monitoring, especially during the survivorship stage [36]. Frontline oncology teams and PCPs manage very high caseloads and navigate broad symptom presentations which, coupled with their lack of psychosocial oncology specialization, means they may be particularly well-served by access to high-quality CPGs compared to specialized clinicians. As such, their absence within the current study represents a gap our knowledge about different clinical needs and challenges to CPG uptake across the cancer care continuum. Furthermore, the survey was administered in English, which does not reflect Canada’s linguistic diversity. This limitation is particularly relevant to Québec, where a substantial number of clinicians operate primarily in French. Consequently, this may reduce the generalizability of our findings. Finally, the present study lacked data on regional healthcare system resources and policies, which may significantly impact how CPGs are endorsed and implemented. As such, our ability to assess how system-level differences across provinces and institutions affect individual clinician behavior is limited. Nevertheless, our findings are informative when keeping limitations in mind.

## Figures and Tables

**Table 1:** Sample Demographic Characteristics (*N* = 172).

Variable	M (SD), Range	*N* (%)
Age	44.85 (12.44), 23–80	
Sex/Gender		
Female		153 (88.95)
Male		15 (8.72)
Non-binary		1 (0.58)
Prefer not to say		3 (1.74)
Highest level of education completed		
Secondary (high) school or equivalent degree		1 (0.58)
Professional apprenticeship or vocational training degree		2 (1.16)
College/CEGEP degree		3 (1.74)
Bachelor’s or equivalent undergraduate university degree		33 (19.19)
Master’s or equivalent second cycle university degree		95 (55.23)
Doctoral or equivalent third cycle university degree		38 (22.09)
Province of practice		
Alberta		14 (8.14)
British Columbia		10 (5.81)
Manitoba		4 (2.33)
New Brunswick		4 (2.33)
Newfoundland and Labrador		2 (1.16)
Nova Scotia		21 (12.21)
Ontario		90 (52.33)
Prince Edward Island		1 (0.58)
Quebec		19 (11.05)
Saskatchewan		7 (4.07)
Profession		
Psychologist		32 (18.60)
Psychotherapist		40 (23.26)
Physicians		3 (1.74)
Nurse		28 (16.28)
Social worker		52 (30.23)
Other (e.g., counsellor, psychoanalyst)		17 (9.88)
Place of employment **		
Hospital		55 (31.98)
University/academic center		17 (9.88)
Specialized/outpatient cancer center		21 (12.21)
Palliative care center		5 (2.91)
Community center		13 (7.56)
Private group/solo practice		100 (58.14)
Other		13 (7.56)
Activities, in portion adding to 100%		
Clinical work (e.g., interventions, treatment planning, record keeping)	63.85 (27.01), 0–100	
Research (e.g., consuming/producing research, training)	13.08 (14.72), 0–76	
Management or administration (e.g., program development, team meetings)	17.00 (19.26), 0–100	
Experience in PSO *, in years		
Less than one year		6 (3.49)
1–3 years		30 (17.44)
3–5 years		24 (13.95)
5–10 years		51 (29.65)
10+ years		61 (35.47)
Time spent providing direct PSO * care per week		
1–10 h		93 (54.07)
11–20 h		28 (16.28)
21–30 h		21 (12.21)
31–40 h		26 (15.12)
>40 h		4 (2.33)

* Note. PSO refers to psychosocial oncology. ** Note 2. Participants instructed to select all that apply.

**Table 2:** Means, Standard Deviations, and Frequencies of Barriers Reported by Clinicians (*N* = 149).

Barriers	M (SD)	Agree and Strongly Agree * n (%)	Disagree and Strongly Disagree * n (%)
*Knowledge*			
1. Psychosocial oncology guidelines are not easily accessible.	3.00 (0.99)	47 (31.54)	43 (28.86)
2. There are too many psychosocial oncology guidelines to choose from.	3.30 (0.94)	69 (46.31)	28 (18.79)
3. It is difficult to know what psychosocial oncology guidelines are available.	3.38 (1.06)	85 (57.05)	30 (20.13)
4. I do not feel confident in my ability to use psychosocial oncology guidelines.	2.48 (1.02)	27 (18.12)	87 (58.39)
5. I do not feel confident in my ability to apply the recommendations of psychosocial oncology guidelines within my practice.	2.42 (0.97)	24 (16.11)	90 (60.40)
6. I did not receive adequate training on how to use and interpret guidelines during my training and education	3.26 (1.23)	72 (48.32)	46 (30.87)
*Evidence and Development*			
7. Psychosocial oncology guideline recommendations are developed based on limited or low-quality evidence.	2.48 (0.80)	9 (6.04)	67 (44.97)
8. I don’t know enough about the quality of currently available psychosocial oncology guidelines.	3.22 (1.13)	67 (44.97)	35 (23.49)
9. Different psychosocial oncology guidelines provide conflicting recommendations.	2.80 (0.72)	17 (11.41)	38 (25.50)
10. The recommendations of psychosocial oncology guidelines are outdated or based on outdated evidence.	2.75 (0.73)	13 (8.72)	40 (26.85)
11. There is a lack of transparency about the development and funding of psychosocial oncology guidelines.	2.84 (0.75)	22 (14.77)	39 (26.17)
12. I disagree with the interventions and/or tools recommended by psychosocial oncology guidelines.	2.56 (0.83)	11 (7.38)	55 (36.91)
*Scope and Relevance*			
13. Psychosocial oncology guideline recommendations are overly simplistic and/or rigid and fail to account for complex clinical case presentations.	3.11 (0.91)	47 (31.54)	33 (22.15)
14. Psychosocial oncology guidelines do not account for the local healthcare system and/or context.	3.49 (0.91)	74 (49.66)	17 (11.41)
15. There are symptoms and/or concerns frequently experienced by patients that are not addressed by psychosocial oncology guidelines.	3.36 (0.86)	63 (42.28)	18 (12.08)
16. Psychosocial oncology guidelines fail to account for the characteristics and preferences of patients.	3.34 (0.87)	60 (40.27)	20 (13.42)
17. The interventions recommended within psychosocial oncology guidelines are not described in enough detail to be helpful (e.g., number of sessions, session topics, etc.).	3.08 (0.81)	40 (26.85)	31 (20.81)
18. Psychosocial oncology guidelines do not help improve patient outcomes.	2.56 (0.86)	13 (8.72)	65 (43.62)
*Feasibility*			
19. I don’t have time to find and use psychosocial oncology guidelines.	3.09 (1.13)	62 (41.61)	49 (32.89)
20. I don’t have time to learn new interventions and/or procedures.	2.75 (1.07)	41 (27.52)	70 (46.98)
21. There are not enough clinical tools or knowledge translation tools to help me implement the recommendations.	2.94 (0.88)	39 (26.17)	49 (32.89)
22. The recommendations of psychosocial oncology guidelines are too costly to implement.	2.77 (0.74)	19 (12.75)	48 (32.21)
23. There is little institutional support for implementing psychosocial oncology guideline recommendations (e.g., training programs, funding).	3.57 (1.00)	79 (53.02)	17 (11.41)
24. The processes at my place of practice are rigid and/or difficult to change.	2.52 (1.20)	34 (22.82)	79 (53.02)
25. The recommendations of psychosocial oncology guidelines do not align with the theoretical orientation or treatment philosophy of my place of practice.	2.60 (0.98)	18 (12.08)	64 (42.95)

* Note. Response options (4 = agree and 5 = strongly agree) were merged to reflect participant agreement with each barrier ranging from 1 to 4.3.4. What Would Encourage CPG use Among Clinicians?

**Table 3:** Use and Perceptions of Currently Existing CPGs Across PSO Clinicians (*N* = 172).

Title of the CPG (Year) *Name of the Development Organization*	Awareness *	Use	Relevance	Perceived Quality
	N (%)	M (SD)	M (SD)	M (SD)
1. Colorectal Cancer Survivorship Care (2015) *American Cancer Society (ACS)* [57]	26 (15.12)	2.52 (1.29)	3.00 (1.05)	3.43 (1.21)
2. Head and Neck Cancer Survivorship Care (2016) *American Cancer Society (ACS)* [58]	18 (10.47)	2.20 (1.08)	2.47 (1.30)	3.20 (1.52)
3. Prostate Cancer Survivorship Care (2014) *American Cancer Society (ACS)* [59]	23 (13.37)	2.11 (0.76)	2.39 (1.24)	3.11 (1.32)
4. Evidence-Based Use of Integrative Therapies During and After Breast Cancer Treatment (2017) *American Cancer Society (ACS)* [60]	20 (11.63)	2.73 (1.03)	2.87 (1.06)	3.00 (1.00)
5. Breast Cancer Survivorship Care (2015) *American Cancer Society (ACS) & American Society of Clinical Oncology (ASCO)* [61]	30 (17.44)	2.62 (1.14)	2.96 (1.20)	3.46 (1.22)
6. Integration of Palliative Care into Standard Oncology Care (2017) *American Society of Clinical Oncology (ASCO)* [62]	35 (20.35)	2.86 (1.18)	3.29 (1.24)	3.54 (1.11)
7. Management of Anxiety and Depression in Adult Survivors of Cancer (2023) *American Society of Clinical Oncology (ASCO)* [63]	31 (18.02)	3.00 (1.20)	3.65 (0.98)	3.81 (0.94)
8. Practical Assessment and Management of Vulnerabilities in Older Patients Receiving Chemotherapy (2023) *American Society of Clinical Oncology (ASCO)* [64]	10 (5.81)	2.86 (1.22)	3.29 (1.38)	3.86 (1.22)
9. Palliative and End-of-Life Care in Lung Cancer (2013) *American College of Chest Physicians* [65]	6 (3.49)	2.50 (1.29)	2.50 (1.29)	3.25 (1.71)
10. Screening, Assessment, and Management of Cancer-Related Fatigue in Adults (2015) *Canadian Association of Psychosocial Oncology (CAPO)* [66]	76 (44.19)	3.21 (1.40)	3.51 (1.37)	3.89 (1.20)
11. Screening, Assessment and Management of Psychosocial Distress, Depression and Anxiety in Adults with Cancer (2015) *Canadian Association of Psychosocial Oncology (CAPO)* [67]	84 (48.84)	3.30 (1.37)	3.58 (1.26)	3.87 (1.12)
12. The Management of Depression in Patients with Cancer (2015) *Cancer Care Ontario (CCO)* [68]	80 (46.51)	3.05 (1.31)	3.36 (1.27)	3.76 (1.18)
13. Follow-up Care and Psychosocial Needs of Survivors of Prostate Cancer (2015) *Cancer Care Ontario (CCO)* [69]	47 (27.33)	2.44 (1.33)	2.72 (1.44)	3.30 (1.39)
14. Interventions to Address Sexual Problems in People with Cancer (2016) *Cancer Care Ontario (CCO)* [70]	40 (23.26)	2.63 (1.30)	2.87 (1.34)	3.39 (1.31)
15. Anxiety and Depression in Adult Cancer Patients (2023) *European Society for Medical Oncology (ESMO)* [71]	12 (6.98)	3.20 (1.48)	3.50 (1.35)	4.30 (0.82)
16. Care of the Adult Cancer Patient at the End of Life (2021) *European Society for Medical Oncology (ESMO)* [72]	10 (5.81)	3.50 (0.76)	3.75 (1.17)	4.25 (0.89)
17. Cancer-Related Fatigue: ESMO Clinical Practice Guidelines for Diagnosis and Treatment (2020) *European Society for Medical Oncology (ESMO)* [73]	9 (5.23)	3.50 (0.54)	3.75 (0.71)	3.88 (0.64)
18. Psychosocial Care for Adult Cancer Patients (2021) *Italian Medical Oncology Association* [74]	6 (3.49)	2.50 (0.58)	2.50 (0.58)	3.50 (1.29)
19. Adolescent and Young Adult (AYA) Oncology, Version 3.2023 (2023) *National Comprehensive Cancer Network (NCCN)* [75]	29 (16.86)	3.23 (1.18)	3.27 (1.31)	3.69 (1.23)
20. Cancer-Related Fatigue, Version 2.2023 (2023) *National Comprehensive Cancer Network (NCCN)* [76]	28 (16.28)	3.74 (1.29)	3.91 (1.16)	4.09 (1.16)
21. Distress Management, Version 2.2023 (2023) *National Comprehensive Cancer Network (NCCN)* [77]	29 (16.86)	3.27 (1.42)	3.50 (1.41)	3.77 (1.19)
22. Palliative Care, Version 1.2023 (2023) *National Comprehensive Cancer Network (NCCN)* [78]	30 (17.44)	3.35 (1.43)	3.52 (1.44)	4.04 (1.22)
23. Survivorship, Version 1.2022 (2022) *National Comprehensive Cancer Network (NCCN)* [79]	28 (16.28)	3.52 (1.20)	3.74 (1.21)	4.17 (0.98)

* Note. N indicates the number of participants who reported familiarity with a CPG. Means and standard deviations for use, relevance, and quality were calculated based on participants who endorsed awareness of the respective CPG, resulting in discrepancies in sample sizes.

**Table 4:** Means, Standard Deviations, and Frequencies of Facilitators Reported by Clinicians (N = 148).

Facilitators	M (SD) Range 1–5	Agree and Strongly Agree * n (%)	Disagree and Strongly Disagree * n (%)
Easily accessible trainings on how to use clinical practice guidelines.	4.07 (0.92)	122 (82.43)	11 (7.43)
2. Easily accessible trainings on the interventions and tools recommended within guidelines.	4.11 (0.86)	126 (85.14)	8 (5.41)
3. Institutional support (e.g., financial support, time off) for attending trainings in guideline use and relevant interventions.	4.11 (1.02)	117 (79.05)	10 (6.76)
4. Access to more patient resources and materials (e.g., webpages, psychoeducational pamphlets, videos, etc.).	3.95 (0.93)	109 (73.65)	10 (6.76)
5. Having access to the quality assessment scores of all currently available psychosocial oncology guidelines.	3.86 (1.01)	107 (72.30)	17 (11.49)
6. More dissemination tools to facilitate use as part of guidelines (e.g., algorithms, flow diagrams).	3.70 (1.02)	85 (57.43)	18 (12.16)
7. Openness of your institution to making changes to procedures and care standards.	3.63 (1.15)	84 (56.76)	20 (13.51)
8. More explicit integration of patient perspectives and preferences as part of guideline development.	4.03 (0.86)	112 (75.68)	5 (3.38)
9. Creation of guideline adaptations for the local context and/or healthcare system.	4.07 (0.84)	115 (77.70)	4 (2.70)
10. Guidelines making the suggested tools and scales more easily accessible (e.g., hyperlinks to scale, provision of different options).	4.07 (0.81)	119 (80.41)	4 (2.70)
11. Widespread endorsement of specific guidelines by colleagues or relevant stakeholders.	3.83 (0.94)	104 (70.27)	12 (8.11)
12. More clear specifications about monitoring and follow-up procedures (e.g., differential treatment recommendations based on scale scores, etc.).	3.86 (0.90)	104 (70.27)	9 (6.08)

* Note. Response options for percentages were merged to reflect participant agreement with each barrier.

## Data Availability

The complete data for the present study is available from the corresponding author upon request.

## Supplementary Materials

The following supporting information can be downloaded at: https://www.mdpi.com/article/10.3390/curroncol33070380/s1, Supplemental Materials S1: Keywords and Search Strategy by Database; Supplemental Materials S2: Canadian Licensing Body Search Portals.

## Appendix A. Survey Development Process and Pilot Testing

This appendix provides additional details on the survey development process and methdology.

### Appendix A.1. Initial Survey Development

**Literature review.** A review of the relevant literature was undertaken to identify existing scales assessing the use, perception, and uptake barriers of CPGs. Several key databases (PsycINFO, MEDLINE, PubMed, and JSTOR) were searched using key terms related to clinical practice guidelines, psychosocial oncology, evidence-based practice, and implementation science. No restrictions were placed on publication year or field. The complete search strategy can be found in the Supplemental Materials.

**Expert consultation.** Following the literature review, two semi-structured interviews were conducted with key stakeholders consisting of clinical experts with leadership roles in Montreal-based psychosocial oncology programs. A summary of the evidence and key themes generated via literature review were provided, and experts were invited to provide feedback on the identified themes. Several key gaps in knowledge were identified: notably, the wide use of non-psychosocial oncology CPGs to guide practice in the symptoms or presentation of trauma due to insufficient research evidence to justify a more targeted CPG. They also highlighted and clarified the barriers to CPG uptake at an organizational level and suggested several barriers be removed or included in the survey. Content from these interviews was used to inform item generation.

**Item development.** Based on the literature review and the themes identified through stakeholder interviews, an initial pool of 174 items was generated collaboratively by two members of the research team (CB and AK). Item generation was informed by attempts to keep language plain and accessible, avoid redundancy, and aligned with constructs measured in related fields. Where possible, items were adapted from existing validated measures to enhance validity and comparability. New items were developed when existing measures did not adequately capture the unique context of psychosocial oncology CPG implementation. The resulting item pool was then organized into key domains that corresponded with the themes identified in the literature and through stakeholder interviews.

### Appendix A.2. Pilot Testing

**Participants and procedure.** Pilot participants were recruited through the professional network of the research team. A total of five participants completed the pilot study. The participants were content experts and experienced clinicians in psychosocial oncology. Four out of five participants had >10 years of experience working in the field and spent >80% of their time devoted to clinical work. Pilot participants were asked to complete the entire survey, with an optional feedback box at the end of each page, as well as an end-of-survey feedback form consisting of a total of five items (4 open-ended and 1 closed-ended items) assessing the clarity and relevance of survey items. Pilot participants who completed the survey and feedback form were sent a $50 Amazon gift card via email as compensation for their time and insight.

**Feeback and item modifications.** Based on pilot study feedback, an open-ended item was created and added to the survey focused on asking participants about gaps in currently available CPGs. Additionally, two demographic questions were modified to allow multiple answers (“*Which of the following best describes your current place of employment?*” and “*Which of the following best describes your current role?*”). Several items throughout the survey received minor modifications to improve their clarity.

## Appendix B. Characteristics of the Final Survey

The final survey was composed of 198 items, of which 115 were conditional, i.e., they were only presented to participants who reported familiarity with the corresponding practice guidelines. Items 1 to 11 pertained to demographic information, including profession, place of employment, experience in years, and time dedicated to psychosocial care. Items 12 to 114 were organized across four key themes: 1. Standards of practice, 2. Awareness, use, and perception of currently available psychosocial oncology CPGs, 3. Barriers and facilitators to CPG use, and 4. Attitude towards evidence-based practice. The complete description of the survey domains and items are below.

***Domain 1: Standards of Practice*** (Items 12–30) is composed of 19 items assessing the standards of clinical practice at participants’ place of employment, including procedures surrounding: screening and assessment, follow-up and monitoring, interventions, and communication and care coordination. These items were derived from the recommendations of currently available CPGs in psychosocial oncology with the goal of assessing how closely the standard practices adhere to CPG recommendations. Items 12 to 29 are scored on a 5-point Likert scale ranging from 1 (*rarely/never*) to 5 (*always/almost always*). Higher scores indicate greater adherence to CPG recommendations. Item 30 is an optional, open-ended question allowing participants to provide additional details about the standards of practice in their place of work if necessary.

***Domain 2: Awareness, Use, and Perception of CPGs*** (Items 31–151) is composed of 120 items evaluating participants’ awareness of currently available psychosocial oncology CPGs. Item 31 consists of a checklist comprising all 23 CPGs identified in a previous study [95]. Participants were asked to select all CPGs they are familiar with. When a CPG was endorsed as familiar in Item 31, participants were directed to complete a set of five items (Items 32–36) scored on a 5-point Likert scale ranging from 1 (*rarely/never*) to 5 (*always/almost always*). Item 32 assesses participants’ use of CPG #1. Item 33 assesses the perceived relevance of the recommendations of CPG #1. Item 34 assesses the perceived quality of CPG #1. Item 35 determines the likelihood participants would recommend CPG #1 for use. Item 36 is an optional, open-ended question inviting participants to elaborate on their use and perception of CPG #1. The same five items were repeated for each of the 23 CPGs, resulting in Items 37 to 146, but only presented to participants for the CPGs endorsed by an individual survey respondent. Items 147 to 149 consist of “*yes*” and “*no*” response options, with a required open-ended response when “*yes*” is endorsed. Item 147 asks about the use of any other psychosocial oncology CPGs not included in the checklist. Item 148 assesses the use of CPGs not specific to psychosocial oncology. Finally, Item 149 asks about potential gaps in psychosocial oncology CPG availability.

***Domain 3: Barriers and Facilitators to CPG Use*** (Items 150–165) is composed of 39 items assessing the barriers and facilitators to CPG use. The items were developed based on studies exploring CPG implementation in psychosocial oncology and broader health-related fields [42,96,97,98], as well as key theoretical frameworks pertaining to implementation, including the Knowledge-to-Action model and Rogers’ Diffusion of Innovations theory. The barriers subscale is composed of 26 items assessing the factors that impede CPG use. Items 150 to 174 were presented in a matrix table with each item consisting of statements about different barriers across four themes: knowledge (Items 150–155), evidence and development (Items 156–161), scope and relevance (Items 162–167), and feasibility (Items 168–174). Response options were based on a 5-point Likert scale ranging from 1 (*strongly disagree*) to 5 (*strongly agree*). Item 175 is an optional, open-ended question inviting participants to elaborate on the barriers to CPG use. The facilitators subscale is composed of 13 items assessing factors that encourage or promote CPG use. Items 176 to 187 were presented in a matrix table with each item consisting of a statement about different facilitators, with response options on a 5-point Likert scale ranging from 1 (*strongly disagree*) to 5 (*strongly agree*). Item 188 is an optional, open-ended question inviting participants to elaborate on these facilitators.

***Domain 4: Attitudes towards Evidence-Based Practice*** (Items 189–198) is composed of a subset of items from the validated Evidence-Based Practice Inventory developed by Kaper et al. (2015) [53]. This scale is composed of 26 items developed to assess adherence to evidence-based practice (EBP) across five dimensions: decision-making, subjective norms, attitudes, perceived behavioral control, and intention and behavior. In the present study, a subset of 10 items was selected by the research team to reduce redundancy and alleviate the burden on participants. Each item was rated on a 7-point Likert scale anchored by contrasting statements (e.g., “*I feel that EBP is useless … useful to improve my patients’ outcomes*”). Higher scores reflect more positive attitudes toward evidence-based practice and greater perceived support for its use in clinical decision-making.

## Appendix C. Detailed Recruitment Strategy

Participant recruitment was conducted in four stages undertaken in parallel. ***Step 1*** involved the distribution of a flyer with a brief description of the study and a QR code to complete the online survey. Physical copies of the flyer were distributed at a national conference for Canadian psychosocial oncology held in Toronto, Canada. ***Step 2*** involved retrieving the contact information of hospitals and community-based clinics offering specialized psychosocial oncology services across Canada through the Canadian Cancer Society’s Community Services Locator, a directory designed to help patients, caregivers, and healthcare professionals find cancer-related services, including emotional support. Targeted emails were sent to these programs and institutions to invite them to distribute the survey among their staff. ***Step 3*** involved retrieving the contact information of individual clinicians who practice within psychosocial oncology. This was conducted through two methods: 1. The online portals of relevant provincial licensing bodies for psychologists and psychotherapists. These portals provide search engines to find licensed professionals and allow for the specifications of areas of practice, including cancer or chronic disease. The licensing bodies for other professionals did not provide a search engine for registered professionals and, thus, could not be included. A complete list of the licensing bodies used for recruitment and search engine key words can be found in the Supplemental Materials; 2. The Psychology Today website (https://www.psychologytoday.com/ca, accessed 1 January 2025) search for each province and territory. The search was specified for ‘therapists’ and ‘cancer’ was selected under the list of specialties. ***Step 4*** involved the creation of a recruitment ad with a brief description of the study and eligibility criteria with a QR code to complete the survey anonymously. This ad was distributed through the newsletters of three organizations: the *Canadian Association of Psychosocial Oncology (CAPO)*, the *Canadian Association of Nurses in Oncology (CANO)*, and the *Canadian Association of Social Workers*.
