# Supplementary material for: Perceptions and Use of Clinical Practice Guidelines in Psychosocial Oncology—A Pan-Canadian Survey of Mental Health and Social Service Professionals
Source: Curr Oncol. 2026 Jun 24;33(7):380. doi: 10.3390/curroncol33070380 (PMC13408611; doi:10.3390/curroncol33070380)
Supplement: Supplementary file 1 [file curroncol-33-00380-s001.zip › curroncol-4262478-supplementary.pdf]

Supplemental Materials S1: Literature Review Search Strategy for Survey Development

Keywords and Search Strategy by Database

Database	Keywords and search strategy
**Medline (1946-)**	(((“Clinical Practice Guidelines as Topic”[MeSH] OR “Guideline Adherence”[MeSH] OR “Practice Guideline”[Publication Type] OR “guideline*”[tiab] OR “clinical practice guideline*”[tiab]) AND (“Psychosocial Oncology” OR “Psycho-Oncology” OR “Oncology”[MeSH] OR “Neoplasms”[MeSH] OR “cancer”[tiab])) AND (“Evidence-Based Practice”[MeSH] OR “Implementation Science” OR “Attitude of Health Personnel”[MeSH] OR “Health Knowledge, Attitudes, Practice”[MeSH] OR “barrier*”[tiab] OR “facilitat*”[tiab] OR “motivator*”[tiab] OR “perception*”[tiab] OR “attitude*”[tiab] OR “use”[tiab] OR “usage”[tiab] OR “utilization”[tiab] OR “uptake”[tiab]))
**PubMed**	((((“clinical practice guideline*”[Title/Abstract]) OR guideline*[Title/Abstract] OR “practice guideline*”[Publication Type])) AND ((psychosocial oncology[Title/Abstract]) OR psycho-oncology[Title/Abstract] OR oncology[Title/Abstract] OR cancer[Title/Abstract])) AND ((“evidence-based practice”[Title/Abstract]) OR “implementation science”[Title/Abstract] OR barrier*[Title/Abstract] OR facilitat*[Title/Abstract] OR motivator*[Title/Abstract] OR perception[Title/Abstract] OR attitude*[Title/Abstract] OR uptake[Title/Abstract] OR use[Title/Abstract] OR utilization[Title/Abstract] OR usage[Title/Abstract]))
PsycINFO (1987-)	((“clinical practice guideline*”.mp OR guideline*.mp OR practice guideline*.mp OR “Guideline Adherence”/exp OR “Professional Practice Guidelines”/exp) AND

	(“psychosocial oncology”.mp OR psycho-oncology.mp OR oncology.mp OR cancer.mp) AND (“evidence-based practice”.mp OR implementation science.mp OR “Implementation”/exp OR “Attitudes”/exp OR barrier*.mp OR facilitat*.mp OR motivator*.mp OR perception.mp OR attitude*.mp OR use.mp OR usage.mp OR utilization.mp OR uptake.mp))
Embase (1974-)	((‘clinical practice guideline’/exp OR ‘guideline adherence’/exp OR ‘practice guideline’/de OR guideline*:ab,ti OR “clinical practice guideline*”:ab,ti) AND (‘psycho-oncology’/exp OR ‘oncology’/exp OR ‘neoplasm’/exp OR ‘cancer’:ab,ti) AND (‘evidence based practice’/exp OR ‘implementation science’/exp OR ‘attitude’/exp OR barrier*:ab,ti OR facilitat*:ab,ti OR motivator*:ab,ti OR perception*:ab,ti OR attitude*:ab,ti OR use:ab,ti OR usage:ab,ti OR utilization:ab,ti OR uptake:ab,ti))
JSTOR	(“clinical practice guideline*” OR guideline* OR “practice guideline*”) AND (“psychosocial oncology” OR “psycho-oncology” OR oncology OR cancer) AND (“evidence-based practice” OR “implementation science” OR barrier* OR facilitat* OR motivator* OR perception OR attitude* OR use OR usage OR utilization OR uptake)

Note. Truncation to find alternative spellings variations is indicated by an asterisk or hyphen after a keyword. An asterisk before a keyword designates the theme as a major topic of the article.

Supplemental Materials S2: Canadian Licensing Body Search Portals

Alberta
College of Alberta Psychologists (CAP)
https://www.cap.ab.ca/
Search directory: https://cap.alinityapp.com/Client/PublicDirectory

British Columbia
British Columbia Psychological Association
https://psychologists.bc.ca/
Search directory: https://psychologists.bc.ca/find-psychologist

Manitoba
The Psychological Association of Manitoba
https://www.cpmb.ca/
Search directory: https://members.mps.ca/

Nova Scotia
Nova Scotia Board of Examiners in Psychology
https://www.nsbep.org/
Search directory: https://www.nsbep.org/public/directory-of-psychologists/

Ontario
College of Psychologists and Behaviour Analysts of Ontario
http://www.cpo.on.ca/
Search directory: https://members.cpbao.ca/public_register/new

Prince Edward Island
Prince Edward Island Psychologists Registration Board
https://www.peipsychology.org/peiprb/
No search directory available

Québec
Ordre des psychologues du Québec
https://www.ordrepsy.qc.ca
Search directory: https://www.ordrepsy.qc.ca/trouver-de-aide

Saskatchewan
Saskatchewan College of Psychologists
http://www.skcp.ca/
Search directory: https://secure.skcp.ca/DirectoryofPsychologists.php

New Brunswick
College of Psychologists of New Brunswick
https://cpnb.ca/en/
Search directory: https://cpnb.ca/en/finding-a-psychologist/
Newfoundland and Labrador
The Newfoundland & Labrador Psychology Board

https://nlpsychboard.ca/
Search directory: https://secure.nlpsychboard.ca/registered_psychologists.php

Northwest Territories
Health and Social Services, Government of Northwest Territories
https://www.hss.gov.nt.ca/en/services/psychologist-licence
No search directory available

Nunavut
Professional Licensing Nunavut Health and Social Services
https://nuphysicians.ca/
No search directory available

Yukon
Not applicable: There is no legislation governing the practice of psychology in Yukon
